# Don’t know much about geography? Decision support for the evaluation of patients with suspected high consequence infectious diseases

**DOI:** 10.1017/ash.2025.10038

**Published:** 2025-09-01

**Authors:** Jacob E. Lazarus, Michelle S. Jerry, Lindsay Germaine, Chloe V. Green, Jason Parente, Eileen F. Searle, Erica S. Shenoy

**Affiliations:** 1 Division of Infectious Diseases, Department of Medicine, Massachusetts General Hospital, Boston, MA, USA; 2 Harvard Medical School, Boston, MA, USA; 3 Clinical Informatics and Digital Health, Mass General Brigham, Boston, MA, USA; 4 Department of Emergency Medicine, Massachusetts General Hospital, Boston, MA, USA; 5 Center for Disaster Medicine, Massachusetts General Hospital, Boston, MA, USA; 6 Infection Control, Mass General Brigham, Boston, MA, USA

## Abstract

EvalHCID is a clinical decision support system integrating outbreak intelligence, symptom onset, and epidemiologic risk factors to identify high consequence infectious diseases (HCIDs) (eg, Ebola). Tested among 20 emergency department (ED) providers, it significantly reduced assessment time, lowered misclassification, and scored “excellent” usability. EvalHCID may improve institutional preparedness and patient outcomes for emerging infectious disease threats.

## Introduction

High consequence infectious diseases (HCIDs) such as Marburg, Ebola, and Lassa fever have the potential for person-to-person spread, high mortality, and limited treatments. Rapid identification and isolation of suspected cases are critical. Yet, failures to isolate patients are common^1^, and nosocomial transmissions have occurred^2^. Because HCID symptoms are nonspecific and overlap with common clinical entities, detailed travel and exposure histories are essential to identify at-risk patients. Healthcare providers require timely access to specialized knowledge including HCID geography, symptoms, incubation periods, and exposure risk factors. However, this expertise is not immediately available to most providers. While isolation of an HCID suspect is critical, isolating patients at low risk for an HCID results in delays in diagnosis and management of more common conditions, resulting in poor patient outcomes^3^.

Clinical decision support systems (CDSS) may facilitate patient assessment, relieving clinician cognitive burden. CDSS has been shown to increase guideline adherence^4^, aid in diagnosing emerging diseases^5^, and assist in infection control precautions^6^. We developed a CDSS, “EvalHCID,” to assist clinicians in categorizing suspect HCID cases as high- or low-risk and performed an initial assessment of its accuracy and usability by emergency department (ED) advanced practice providers (APPs).

## Methods

Infectious diseases, infection prevention, and information technology specialists provided iterative feedback on EvalHCID logic to ensure application of Centers for Disease Control and Prevention (CDC) guidance to a range of scenarios for select viral hemorrhagic fevers, Middle East Respiratory Syndrome, and novel influenza. EvalHCID was integrated into a pre-existing, mandatory ED workflow that includes a universal symptom and international travel screen (Supplementary Figure 1). EvalHCID was incorporated into the electronic health record (Epic Systems) in May 2024.

ED APPs, who provide the majority of the frontline care in our ED, were recruited for an accuracy and usability assessment. Usability was assessed using the System Usability Scale^7^. In a classroom setting, participants evaluated four hypothetical patient scenarios, each emulating a different HCID and focusing on the four core components of an HCID evaluation: travel, symptoms, disease incubation period, and epidemiologic risk (Supplementary Materials). In two scenarios, the provider used curated intranet resources (the previous workflow), and in the other two, EvalHCID. Intranet resources included a list of countries with circulating HCIDs, as well as HCID guides providing a grid of symptoms and exposure risk factors (Supplementary Figure 2). Participants timed their completion of each scenario. This study received a non-human subjects determination by the Mass General Brigham IRB. Statistical analysis used two-tailed t-tests.

## Results

**Figure 1.**
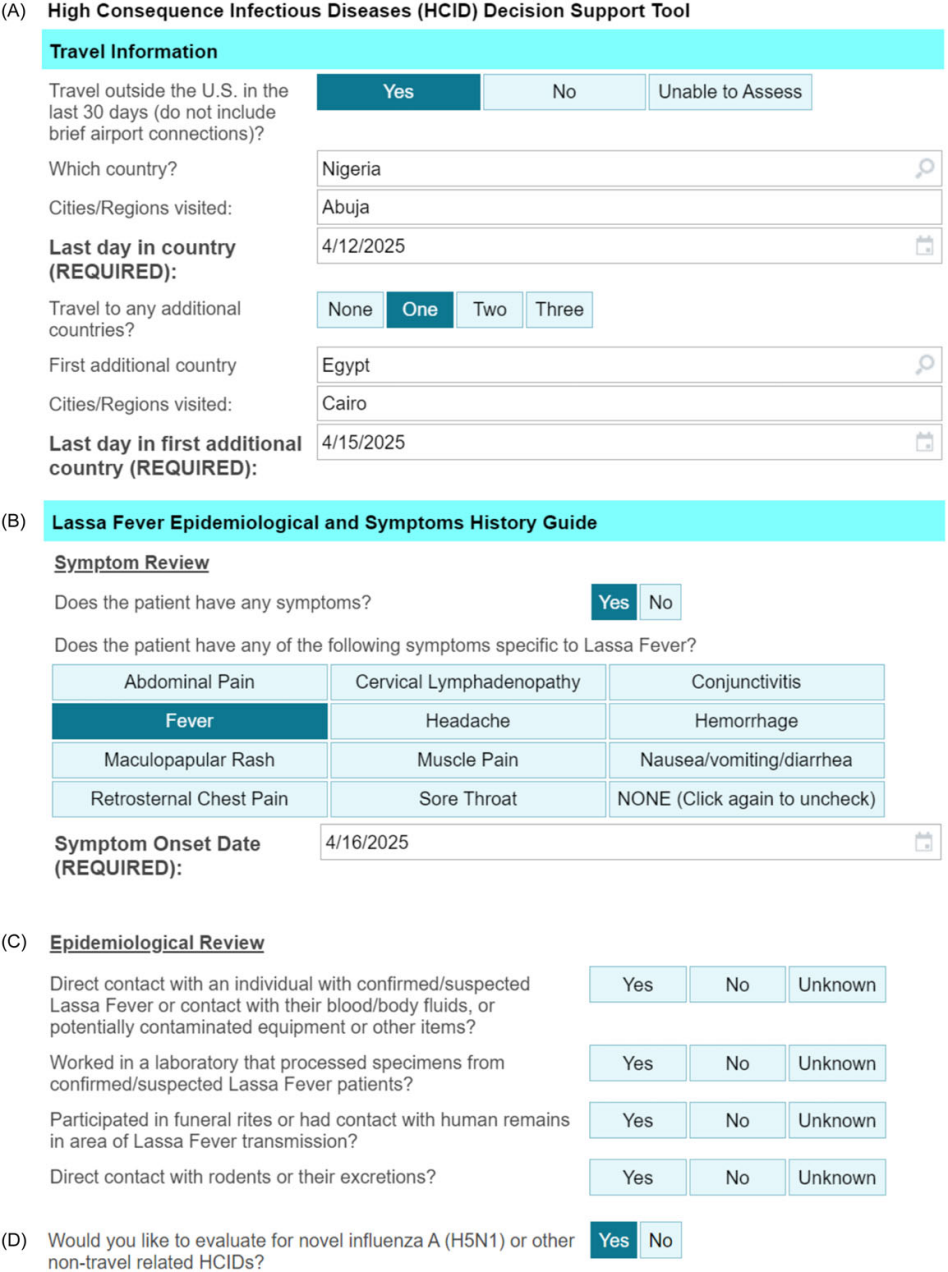
EvalHCID Clinical Decision Support System. A) Travel history is imported automatically from a required universal entry screening, or entered manually by the provider. The last day in-country is recorded. B) Country travel is cross-referenced against an internal, curated database of circulating, high-impact HCIDs, and a review of symptoms focused to circulating HCIDs loads to facilitate history taking. The date of symptom onset is used to check if the patient is within a “plausible incubation period” for the HCID of interest (EvalHCID subtracts the last day in country from the symptom onset day). C) If the patient has a relevant symptom and is within a plausible incubation period, epidemiological and exposure review loads. D) EvalHCID can be used for patients who have not traveled, to assist in the evaluation of domestically acquired HCIDs like novel influenza (such as H5N1). See also Supplementary Movie 1.

Providers launch EvalHCID based on one of the two prompts: a best practice advisory that loads (Supplementary Figure 3) when, in initial screening upon presentation to the ED or inpatient admission, patients report travel to a country with a current outbreak and relevant symptoms of possible infection (Supplementary Figure 1); Providers may also load EvalHCID at-will. To ensure that patients at low-risk for HCIDs do not experience delays in usual evaluation, we designed EvalHCID with a series of diagnostic “off-ramps” (Supplementary Figure 4), with additional risk stratification questions loading only if a patient continues to progressively meet high-risk criteria. Travel history from the initial screening is imported into EvalHCID or entered manually, with support for itineraries containing up to four countries (Figure 1A and Supplementary Movie 1). This is referenced against an internal country database, and a symptom review guide specific to the relevant HCID is loaded for travel to countries with current outbreaks (Figure 1B). If the patient reports a relevant symptom, onset date is checked against their last day in-country to determine if symptoms developed within an incubation period consistent with that HCID. If symptoms developed outside this period, EvalHCID assesses the patient as low-risk, while if within that period, epidemiological review loads (Figure 1C). If the patient denies epidemiologic risk factors for that HCID, EvalHCID assesses the patient as low-risk, while if the patient reports a risk factor, EvalHCID assesses the patient as high-risk and provides isolation guidance and infection control contact information. A note is automatically generated in the electronic health record (Supplementary Figure 5). EvalHCID may also be used in the absence of patient travel for the assessment of exposure risk factors for domestically acquired infections (eg, H5N1) (Figure 1D).

Twenty ED APPs were recruited to participate in an analysis of EvalHCID accuracy and usability. Participants had a median age of 35 years (IQR, 33-39) and 5 years (IQR 3-10) in their current roles. They reported previously conducting a median of 1 to 2 HCID risk assessments. Accuracy was assessed in two domains: at-risk for HCID (ie, which HCID the patient was at risk for based on travel) and HCID risk level (high- vs low-risk based on symptom, incubation period, and exposure factors). The rates of misidentification of at-risk for HCID were low using either intranet resources or EvalHCID (2.5% and 5%, respectively). In contrast, use of intranet resources resulted in more frequent misidentification of HCID risk level (45%), compared to 10% risk misclassification by participants using EvalHCID. Per case, the duration of assessment was significantly faster using EvalHCID (3 min versus 5 min, *P* < 0.01). Users reported similar confidence in the accuracy of their evaluation regardless of the method; however, they reported significantly higher perceived difficulty using intranet resources (4 out of 5 on a Likert scale) compared to using EvalHCID (2 out of 5) (*P* < 0.01). Overall, EvalHCID was scored at 90 out of 100 (CI, 86–94) on the System Usability Scale, corresponding to a usability of “Excellent.”

## Discussion

As laid out in the Joint Commission’s standards for HCID or Special Pathogens, healthcare organizations must identify high-risk patients at points of entry^8^. While EvalHCID can be used as a standalone tool toward this aim, it is likely best paired with structured travel screening with a robust workflow to prompt providers of patients with a positive screen to perform EvalHCID. This ensures that high-risk patients are identified rapidly, undergo appropriate isolation, and the appropriate individuals are informed to advise on next steps. In the user analysis, participants using EvalHCID as opposed to conventional resources conducted a faster evaluation and demonstrated substantially less misclassification of patients as being high-risk for an HCID. This is important for multiple reasons: rooms appropriate for high-risk patients are limited in most EDs; extended evaluations of misclassified patients expend significant provider clinical time; and patient experience can be degraded by undergoing unnecessary evaluation for an HCID. Perhaps most importantly, unnecessary HCID evaluations delay the diagnosis of more common HCID mimics. During the 2014–2016 Ebola virus disease outbreak, CDC identified delays in malaria evaluation in travelers from West Africa^3^, and delays in diagnosis have been identified as the unifying factor in fatal malaria cases in the United States^9^.

In summary, we developed and implemented EvalHCID, a CDSS to assist in the identification of returning travelers at high-risk for having an HCID. By providing a structured approach that extends specialized expertise to the frontline clinician, this tool has the potential to improve HCID preparedness, particularly for smaller facilities without infectious disease providers. Limitations of our study include its single-center design and the use of scenarios created specifically for use in the study. Future efforts should further develop and validate scenarios that test core provider HCID evaluation competencies in multiple provider roles.

## Supporting information

10.1017/ash.2025.10038.sm001 Lazarus et al. supplementary material 1 Lazarus et al. supplementary material

10.1017/ash.2025.10038.sm002 Lazarus et al. supplementary material 2 Lazarus et al. supplementary material

## Supplementary material

The supplementary material for this article can be found at https://doi.org/10.1017/ash.2025.10038.

## References

[1] Foote MMK , Styles TS , Quinn CL . Assessment of hospital emergency department response to potentially infectious diseases using unannounced mystery patient drills - New York city, 2016. MMWR Morb Mortal Wkly Rep 2017;66:945–949. 28910268 10.15585/mmwr.mm6636a2 PMC5657916

[2] The Texas Health Presbyterian Hospital Ebola Crisis: A Perfect Storm of Human Errors, System Failures and Lack of Mindfulness. [cited 2024 Dec 17];Available from: https://psnet.ahrq.gov/issue/texas-health-presbyterian-hospital-ebola-crisis-perfect-storm-human-errors-system-failures

[3] Tan KR , Cullen KA , Koumans EH , Arguin PM . Inadequate diagnosis and treatment of malaria among travelers returning from Africa during the Ebola epidemic--United States, 2014-2015. MMWR Morb Mortal Wkly Rep 2016;65:27–29. 26796654 10.15585/mmwr.mm6502a3

[4] Sutton RT , Pincock D , Baumgart DC , Sadowski DC , Fedorak RN , Kroeker KI . An overview of clinical decision support systems: benefits, risks, and strategies for success. NPJ Digit Med 2020;3:17. 32047862 10.1038/s41746-020-0221-y PMC7005290

[5] Lazarus JE , Green CV , Jerry MS , et al. Separating the rash from the chaff: novel clinical decision support deployed during the mpox outbreak. *Infect Control Hosp Epidemiol* 2024;1–3. 10.1017/ice.2024.51 PMC11747935 38561199

[6] Dugdale CM , Rubins DM , Lee H , et al. COVID-19 diagnostic clinical decision support: a pre-post implementation study of CORAL (COvid Risk cALculator). Clin Infect Dis 2021;73:2248–2256. doi: 10.1093/cid/ciab111 33564833 PMC7929052

[7] Chfp ABP , Kortum P , Miller J . Determining What Individual SUS Scores Mean: Adding an Adjective Rating Scale [Internet]. JUX - The Journal of User Experience. 2009 [cited 2024 Nov 20];Available from: https://uxpajournal.org/determining-what-individual-sus-scores-mean-adding-an-adjective-rating-scale/

[8] High-consequence Infectious Diseases or Special Pathogens - Understanding The Requirements (IC.07.01.01) [Internet]. [cited 2024 Nov 22]; Available from: https://www.jointcommission.orghttps://www.jointcommission.org/standards/standard-faqs/critical-access-hospital/infection-prevention-and-control-ic/000002503/

[9] Mace KE . Malaria Surveillance — United States, 2018. MMWR Surveill Summ 2022;71:1–35. Available from: https://www.cdc.gov/mmwr/volumes/71/ss/ss7108a1.htm 10.15585/mmwr.ss7108a1 PMC9470224 36048717

