# Supplementary material for: Don’t know much about geography? Decision support for the evaluation of patients with suspected high consequence infectious diseases
Source: Antimicrob Steward Healthc Epidemiol. 2025 Sep 1;5(1):e192. doi: 10.1017/ash.2025.10038 (PMC12415783; doi:10.1017/ash.2025.10038)
Supplement: Lazarus et al. supplementary material 1 — Lazarus et al. supplementary material [file S2732494X25100387sup001.docx]

**Supplementary Material**

**Table of contents**

Supplementary Figure 1……………………………………………………………………………………2

Supplementary Figure 2……………………………………………………………………………………3

Supplementary Figure 3……………………………………………………………………………………4

Supplementary Figure 4……………………………………………………………………………………5

Supplementary Figure 5……………………………………………………………………………………6

Study Design and Patient Scenarios………………………………………………………………………..7


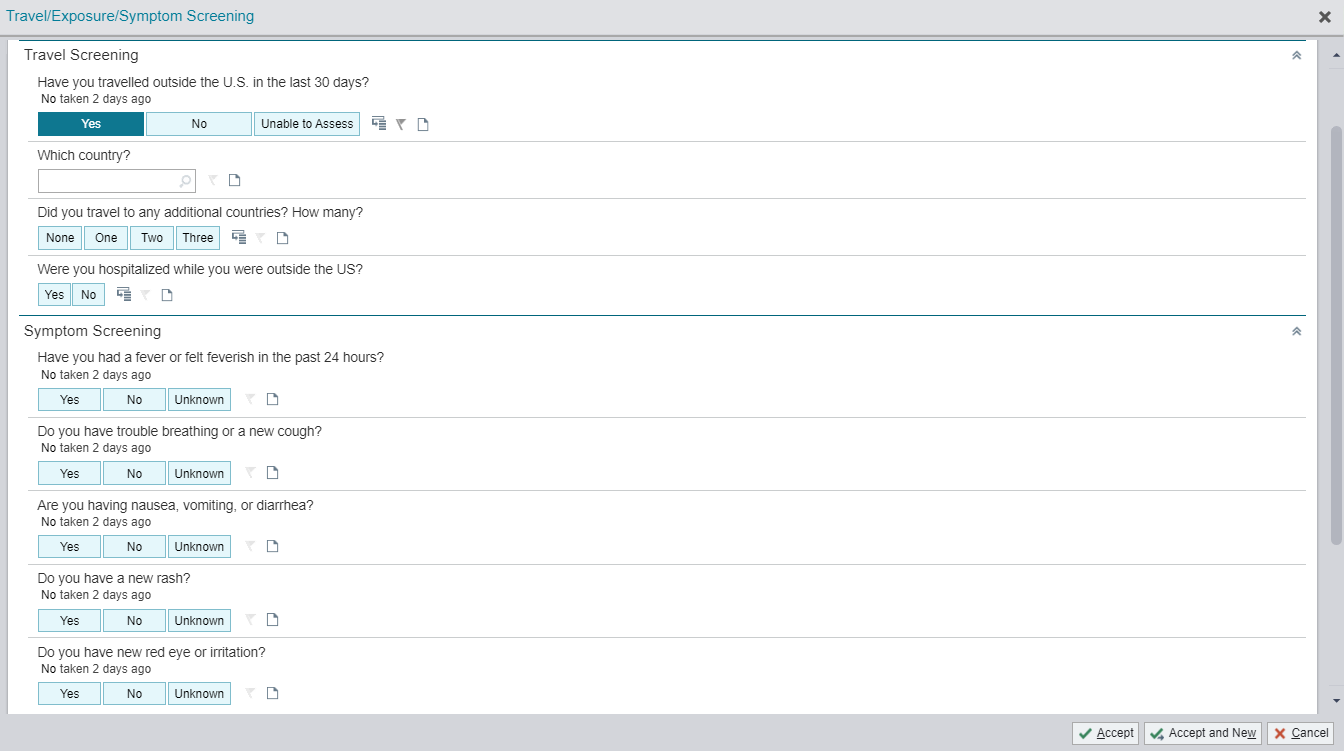
 **Supplementary figure 1 –** A screenshot of our pre-existing, mandatory ED symptom and international travel screen, the results of which may trigger a Best Practice Advisory in the event of a substantial HCID outbreak.


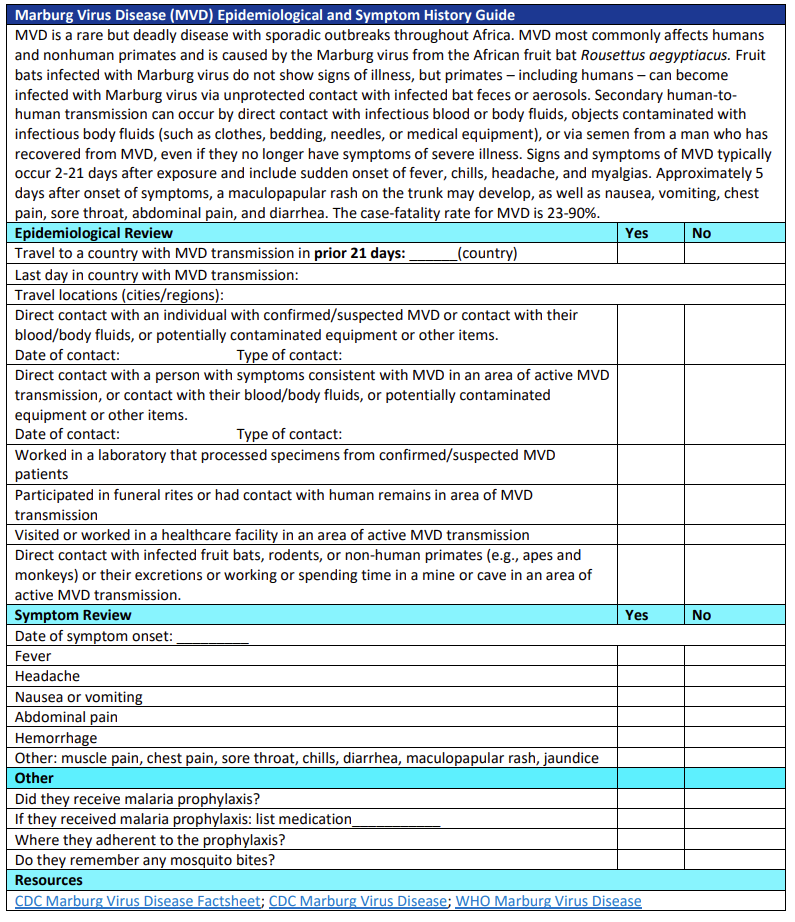


**Supplementary figure 2 -** An example of an HCID symptom and exposure grid available on our intranet.


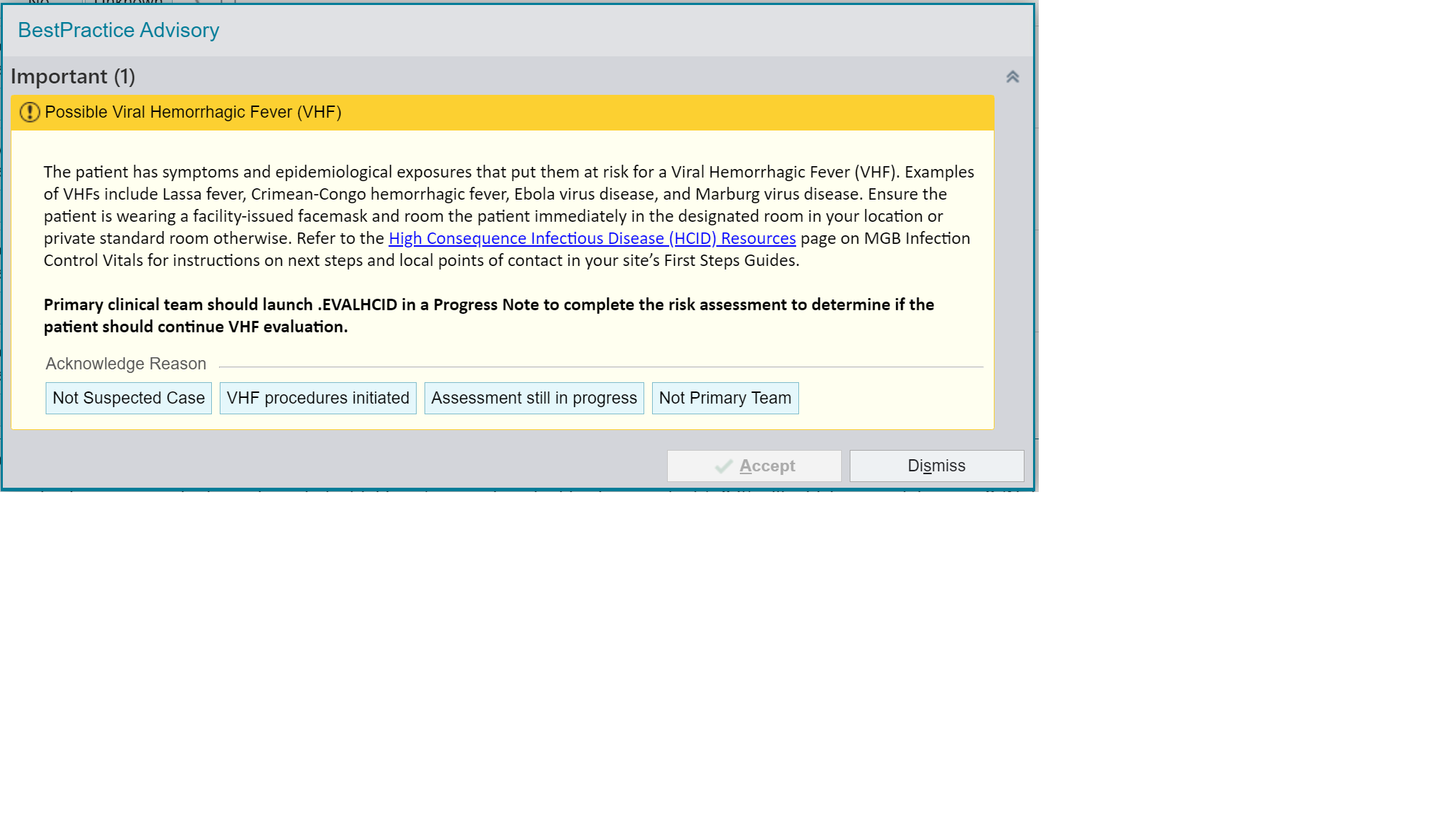
**Supplementary figure 3 -** An example of a viral hemorrhagic fever (VHF) Best Practice Advisory (BPA) when a symptomatic patient screens positive for travel from a country with a circulating HCID.


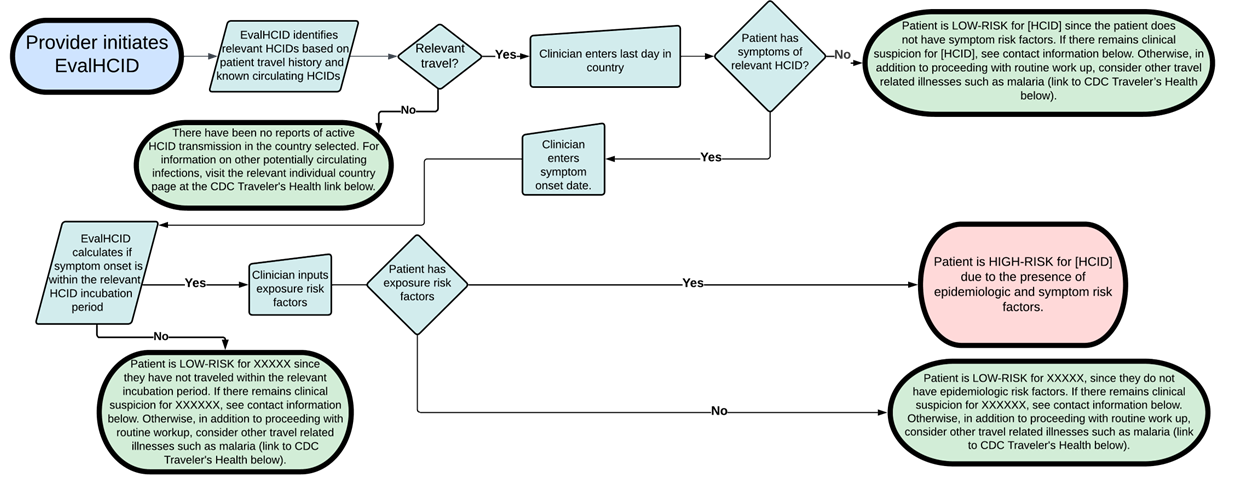


**Supplementary figure 4 -** Logic flow diagram for EvalHCID. To ensure that patients at low-risk for HCIDs do not have their usual evaluation delayed, we designed EvalHCID with a series of diagnostic “off-ramps”, with additional risk stratification questions loading only if a patient continues to progressively meet high-risk criteria.


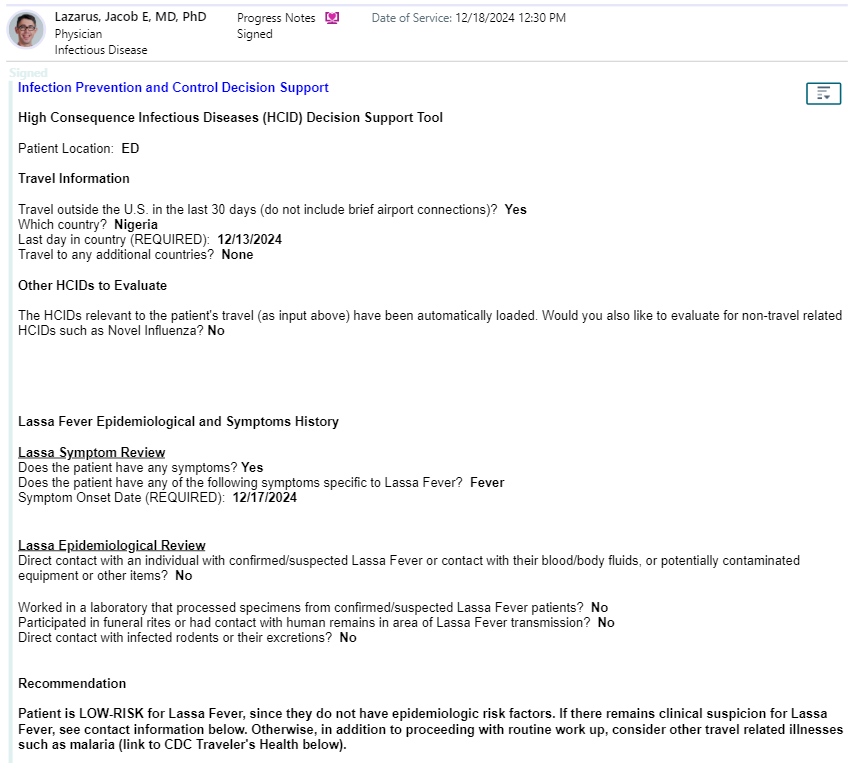
**Supplementary figure 5 -** EvalHCID automatically writes a note, documenting the workup, which is saved into the electronic health record.

## Study Design and Patient Scenarios:

Participants were given a printed study packet containing four hypothetical patient scenarios to evaluate, each including a different High Consequence Infectious Disease (HCID). For each HCID, four variations of scenarios were created to highlight different features of an HCID evaluation (relevant symptoms, an incubation period consistent with the relevant HCID, and epidemiology/exposure risk factors). In two of the four scenarios given to a participant, the participant was instructed to use online intranet resources (Traditional method) and in the other two scenarios, the user was instructed to use the EvalHCID CDS tool. To ensure balance, an equal number of participants started with EvalHCID or online intranet resources to ensure the study was balanced. The starting scenario variation was also randomized within the study packet. Participants were tasked with making two determinations: which HCID the patient should be evaluated for, and if the patient was at high- or low- risk for it. Participants tracked how much time was spent conducting their evaluation and rated on a five-point Likert scale the perceived difficulty and their confidence in the patient evaluation. Scenarios that were evaluated using EvalHCID had an additional page that contained the System Usability Scale (SUS) for the participant to complete.

## Instructions Given to Users:

1. Complete this packet in order, from the 1^st^ page to the last.
2. You will evaluate 4 test patients for High Consequence Infectious Diseases (HCIDs). Each scenario will be printed on its own piece of paper.
3. You will be asked to decide whether a patient is “low-risk” or “high-risk” for HCID(s). We define these terms as:
4. Low-risk: Very unlikely to have an HCID. Can proceed with the usual workup under Standard Precautions and addition of Transmission-Based Precautions as appropriate (e.g., patient on Contact Precautions for MRSA).
5. High-risk: HCID possible. The patient remains isolated, and Biothreats MD should be contacted to perform additional risk stratification.
6. To accomplish this risk determination, you will use one of two methods:
7. The traditional method, referring to information on Vitals and other resources as necessary.
8. Using .evalHCID.
9. Before beginning a scenario, start a timer on your phone to see how long the scenario takes you.
10. We will provide an MRN of a test patient in which to either:
11. Start a documentation encounter and write a short note (a sentence or two) explaining your thought process why the patient is either low-risk or high-risk.
12. Start a documentation encounter and use .evalHCID.
13. You have the option of consulting with Biothreats MD (available outside the computer room) if you have difficulty determining if the patient is low-risk or high-risk. This is not required for either of the two methods of evaluation.
14. After you sign your note, record the time it took on the bottom of the piece of paper.
15. Short surveys will be included throughout the packet. Complete them as you encounter them. You may leave after you have completed the surveys. Don’t forget your gift card! Thank you for your help improving the .evalHCID SmartPhrase.

## System Usability Scale:

|  | Strongly Disagree | Somewhat Disagree | Neutral | Somewhat Agree | Strongly Agree |
| --- | --- | --- | --- | --- | --- |
| 1. I think I would like to use this tool frequently. | □ | □ | □ | □ | □ |
| 1. I found the tool unnecessarily complex. | □ | □ | □ | □ | □ |
| 1. I thought the tool was easy to use. | □ | □ | □ | □ | □ |
| 1. I think that I would need the support of a technical person to be able to use this system. | □ | □ | □ | □ | □ |
| 1. I found the various functions in this tool worked well together. | □ | □ | □ | □ | □ |
| 1. I thought there was too much inconsistency in this tool. | □ | □ | □ | □ | □ |
| 1. I would imagine that most people would learn to use this tool very quickly. | □ | □ | □ | □ | □ |
| 1. I found the tool very difficult to use. | □ | □ | □ | □ | □ |
| 1. I felt very confident using the tool. | □ | □ | □ | □ | □ |
| 1. I needed to learn a lot of things before I could get going with this tool. | □ | □ | □ | □ | □ |

Did you experience any technical difficulties during your use of .evalHCID?

- ​​Yes
- ​​No

What about .evalHCID could be improved? Please write in your suggestions below:

**Scenario A1**

A 27-year-old man presents to the emergency department 8 days after returning from visiting family in Pakistan.

He endorses worsening cough associated with shortness of breath that he first appreciated 3 days ago. There is no associated fever or feverishness. He has had no known sick contacts.

He spent the majority of his time with family in Rural Balochistan. His grandmother raises sheep. He does not report finding any ticks on himself but did pull several off the family dog.

He did not attend any funerals, nor was he admitted to a hospital. He works as a paralegal and in his spare time is training to be a docent at the New Bedford Whaling Museum.

Complete this scenario using: **[EVALHCID/ TRADITIONAL METHOD]**

How much time did you spend to complete this scenario?  _______ minutes

Which, HCID(s) (if any) did you evaluate the patient for?   _____________________________

In your assessment, is the patient low-risk or high-risk for the above HCID(s)?

- ​​Low-risk (No further workup needed)
- ​​High-risk (Contact Biothreats)

|  | Strongly Disagree | Somewhat Disagree | Neutral | Somewhat Agree | Strongly Agree |
| --- | --- | --- | --- | --- | --- |
| 1. It was difficult to determine if the patient was high-risk for an HCID. | □ | □ | □ | □ | □ |
| 1. I am confident in my determination. | □ | □ | □ | □ | □ |

**Scenario A2**

A 27-year-old man presents to the emergency department 8 days after returning from visiting family in Pakistan.

He endorses worsening feverishness and headache that he first appreciated 3 days ago. He has had no known sick contacts.

He spent the majority of his time with family in rural Balochistan. His grandmother raises sheep. He does not report finding any ticks on himself but did pull several off the family dog.

He did not attend any funerals, nor was he admitted to a hospital. He works as a paralegal and in his spare time is training to be a docent at the New Bedford Whaling Museum.

Complete this scenario using: **[EVALHCID/ TRADITIONAL METHOD]**

How much time did you spend to complete this scenario?  _______ minutes

Which, HCID(s) (if any) did you evaluate the patient for?   _____________________________

In your assessment, is the patient low-risk or high-risk for the above HCID(s)?

- ​​Low-risk (No further workup needed)
- ​​High-risk (Contact Biothreats)

|  | Strongly Disagree | Somewhat Disagree | Neutral | Somewhat Agree | Strongly Agree |
| --- | --- | --- | --- | --- | --- |
| 1. It was difficult to determine if the patient was high-risk for an HCID. | □ | □ | □ | □ | □ |
| 1. I am confident in my determination. | □ | □ | □ | □ | □ |

**Scenario A3**

A 27-year-old man presents to the emergency department 25 days after returning from visiting family in Pakistan.

He endorses worsening feverishness and headache that he first appreciated 2 days ago. He has had no known sick contacts.

He spent the majority of his time with family in rural Balochistan. His grandmother raises sheep. He does not report finding any ticks on himself but did pull several off the family dog.

He did not attend any funerals, nor was he admitted to a hospital. He works as a paralegal and in his spare time is training to be a docent at the New Bedford Whaling Museum.

Complete this scenario using: **[EVALHCID/ TRADITIONAL METHOD]**

How much time did you spend to complete this scenario?  _______ minutes

Which, HCID(s) (if any) did you evaluate the patient for?   _____________________________

In your assessment, is the patient low-risk or high-risk for the above HCID(s)?

- ​​Low-risk (No further workup needed)
- ​​High-risk (Contact Biothreats)

|  | Strongly Disagree | Somewhat Disagree | Neutral | Somewhat Agree | Strongly Agree |
| --- | --- | --- | --- | --- | --- |
| 1. It was difficult to determine if the patient was high-risk for an HCID. | □ | □ | □ | □ | □ |
| 1. I am confident in my determination. | □ | □ | □ | □ | □ |

**Scenario A4**

A 27-year-old man presents to the emergency department 8 days after returning from visiting family in Pakistan.

He endorses worsening feverishness and headache that he first appreciated 2 days ago. He has had no known sick contacts.

He spent the majority of his time with family in urban Lahore. He reports no unusual exposures, though he did visit a nearby water park 2 days before flying home.

He did not attend any funerals, nor was he admitted to a hospital. He works as a paralegal and in his spare time is training to be a docent at the New Bedford Whaling Museum.

Complete this scenario using: **[EVALHCID/ TRADITIONAL METHOD]**

How much time did you spend to complete this scenario?  _______ minutes

Which, HCID(s) (if any) did you evaluate the patient for?   _____________________________

In your assessment, is the patient low-risk or high-risk for the above HCID(s)?

- ​​Low-risk (No further workup needed)
- ​​High-risk (Contact Biothreats)

|  | Strongly Disagree | Somewhat Disagree | Neutral | Somewhat Agree | Strongly Agree |
| --- | --- | --- | --- | --- | --- |
| 1. It was difficult to determine if the patient was high-risk for an HCID. | □ | □ | □ | □ | □ |
| 1. I am confident in my determination. | □ | □ | □ | □ | □ |

**Scenario B1**

A 52-year-old man presents to the emergency department 8 days after returning from visiting family in Saudi Arabia.

He reports a worsening rash that he first appreciated 3 days ago. It started as an area of localized redness on his left foot, but over the course of the last day, it has spread up his leg. He reports that there is a dull, aching pain. He denies fevers or feverishness at home. He has had no known sick contacts.

He spent the majority of his time with family in urban Riyadh though he visited a cousin in the countryside who manages a herd of camel. They shared a traditional meal of Margoogat Lahm and camel milk.

He did not attend any funerals, nor was he admitted to a hospital. He works at the Boston Aquarium but has no direct animal contact there.

Complete this scenario using: **[EVALHCID/ TRADITIONAL METHOD]**

How much time did you spend to complete this scenario?  _______ minutes

Which, HCID(s) (if any) did you evaluate the patient for?   _____________________________

In your assessment, is the patient low-risk or high-risk for the above HCID(s)?

- ​Low-risk (No further workup needed)
- ​​High-risk (Contact Biothreats)

|  | Strongly Disagree | Somewhat Disagree | Neutral | Somewhat Agree | Strongly Agree |
| --- | --- | --- | --- | --- | --- |
| 1. It was difficult to determine if the patient was high-risk for an HCID. | □ | □ | □ | □ | □ |
| 1. I am confident in my determination. | □ | □ | □ | □ | □ |

**Scenario B2**

A 52-year-old man presents to the emergency department 9 days after returning from visiting family in Saudi Arabia.

He reports a worsening fever and shortness of breath that he first appreciated 2 days ago. He has had no known sick contacts.

He spent the majority of his time with family in urban Riyadh though he visited a cousin in the countryside who manages a herd of camel. They shared a traditional meal of Margoogat Lahm and camel milk.

He did not attend any funerals, nor was he admitted to a hospital. He works at the Boston Aquarium but does not have any direct animal contact there.

Complete this scenario using: **[EVALHCID/ TRADITIONAL METHOD]**

How much time did you spend to complete this scenario?  _______ minutes

Which, HCID(s) (if any) did you evaluate the patient for?   _____________________________

In your assessment, is the patient low-risk or high-risk for the above HCID(s)?

- ​​Low-risk (No further workup needed)
- ​​High-risk (Contact Biothreats)

|  | Strongly Disagree | Somewhat Disagree | Neutral | Somewhat Agree | Strongly Agree |
| --- | --- | --- | --- | --- | --- |
| 1. It was difficult to determine if the patient was high-risk for an HCID. | □ | □ | □ | □ | □ |
| 1. I am confident in my determination. | □ | □ | □ | □ | □ |

**Scenario B3**

A 52-year-old man presents to the emergency department 19 days after returning from visiting family in Saudi Arabia.

He reports a worsening fever and shortness of breath that he first appreciated 2 days ago. He has had no known sick contacts.

He spent the majority of his time with family in urban Riyadh though he visited a cousin in the countryside who manages a herd of camel. They shared a traditional meal of Margoogat Lahm and camel milk.

He did not attend any funerals, nor was he admitted to a hospital. He works at the Boston Aquarium but does not have any direct animal contact there.

Complete this scenario using: **[EVALHCID/ TRADITIONAL METHOD]**

How much time did you spend to complete this scenario?  _______ minutes

Which, HCID(s) (if any) did you evaluate the patient for?   _____________________________

In your assessment, is the patient low-risk or high-risk for the above HCID(s)?

- ​Low-risk (No further workup needed)
- ​​High-risk (Contact Biothreats)

|  | Strongly Disagree | Somewhat Disagree | Neutral | Somewhat Agree | Strongly Agree |
| --- | --- | --- | --- | --- | --- |
| 1. It was difficult to determine if the patient was high-risk for an HCID. | □ | □ | □ | □ | □ |
| 1. I am confident in my determination. | □ | □ | □ | □ | □ |

**Scenario B4**

A 52-year-old man presents to the emergency department 8 days after returning from visiting family in Saudi Arabia.

He reports a worsening fever and shortness of breath that he first appreciated 2 days ago. He has had no known sick contacts.

He spent the majority of his time with family in urban Riyadh though did have a one-day excursion into the desert where he went dune-bashing. He had no camel contact.

He did not attend any funerals, nor was he admitted to a hospital. He works at the Boston Aquarium but does not have any direct animal contact there.

Complete this scenario using: **[EVALHCID/ TRADITIONAL METHOD]**

How much time did you spend to complete this scenario?  _______ minutes

Which, HCID(s) (if any) did you evaluate the patient for?   _____________________________

In your assessment, is the patient low-risk or high-risk for the above HCID(s)?

- ​​Low-risk (No further workup needed)
- ​​High-risk (Contact Biothreats)

|  | Strongly Disagree | Somewhat Disagree | Neutral | Somewhat Agree | Strongly Agree |
| --- | --- | --- | --- | --- | --- |
| 1. It was difficult to determine if the patient was high-risk for an HCID. | □ | □ | □ | □ | □ |
| 1. I am confident in my determination. | □ | □ | □ | □ | □ |

**Scenario C1**

A 71-year-old woman presents to the emergency department 8 days after returning from visiting family in Nigeria.

She reports worsening cough and shortness of breath that she first appreciated 3 days ago. She denies fevers or feverishness or other systemic symptoms. She has had no known sick contacts.

She spent the majority of her time visiting family in the Yoruba region. She stayed in a traditional thatched home. She denies directly seeing any rodents, though she did notice rodent droppings and her sister was complaining about a recent infestation.

She did not attend any funerals, nor was she admitted to a hospital. She retired recently after working in the Somerville school district for many years.

Complete this scenario using: **[EVALHCID/ TRADITIONAL METHOD]**

How much time did you spend to complete this scenario?  _______ minutes

Which, HCID(s) (if any) did you evaluate the patient for?   _____________________________

In your assessment, is the patient low-risk or high-risk for the above HCID(s)?

- ​​Low-risk (No further workup needed)
- ​​High-risk (Contact Biothreats)

|  | Strongly Disagree | Somewhat Disagree | Neutral | Somewhat Agree | Strongly Agree |
| --- | --- | --- | --- | --- | --- |
| 1. It was difficult to determine if the patient was high-risk for an HCID. | □ | □ | □ | □ | □ |
| 1. I am confident in my determination. | □ | □ | □ | □ | □ |

**Scenario C2**

A 71-year-old woman presents to the emergency department 8 days after returning from visiting family in Nigeria.

She reports the relatively rapid onset of fever and abdominal pain over the prior 2 days. She denies cough or shortness of breath. She has had no known sick contacts.

She spent the majority of her time visiting family in the Yoruba region. She stayed in a traditional thatched home. She denies directly seeing any rodents, though she did notice rodent droppings and her sister was complaining about a recent infestation.

She did not attend any funerals, nor was she admitted to a hospital. She retired recently after working in the Somerville school district for many years.

Complete this scenario using: **[EVALHCID/ TRADITIONAL METHOD]**

How much time did you spend to complete this scenario?  _______ minutes

Which, HCID(s) (if any) did you evaluate the patient for?   _____________________________

In your assessment, is the patient low-risk or high-risk for the above HCID(s)?

- ​​Low-risk (No further workup needed)
- ​​High-risk (Contact Biothreats)

|  | Strongly Disagree | Somewhat Disagree | Neutral | Somewhat Agree | Strongly Agree |
| --- | --- | --- | --- | --- | --- |
| 1. It was difficult to determine if the patient was high-risk for an HCID. | □ | □ | □ | □ | □ |
| 1. I am confident in my determination. | □ | □ | □ | □ | □ |

**Scenario C3**

A 71-year-old woman presents to the emergency department 25 days after returning from visiting family in Nigeria.

She reports the relatively rapid onset of fever and abdominal pain over the prior 2 days. She denies cough or shortness of breath. She has had no known sick contacts.

She spent the majority of her time visiting family in the Yoruba region. She stayed in a traditional thatched home. She denies directly seeing any rodents, though she did notice rodent droppings and her sister was complaining about a recent infestation.

She did not attend any funerals, nor was she admitted to a hospital. She retired recently after working in the Somerville school district for many years.

Complete this scenario using: **[EVALHCID/ TRADITIONAL METHOD]**

How much time did you spend to complete this scenario?  _______ minutes

Which, HCID(s) (if any) did you evaluate the patient for?   _____________________________

In your assessment, is the patient low-risk or high-risk for the above HCID(s)?

- ​​Low-risk (No further workup needed)
- ​​High-risk (Contact Biothreats)

|  | Strongly Disagree | Somewhat Disagree | Neutral | Somewhat Agree | Strongly Agree |
| --- | --- | --- | --- | --- | --- |
| 1. It was difficult to determine if the patient was high-risk for an HCID. | □ | □ | □ | □ | □ |
| 1. I am confident in my determination. | □ | □ | □ | □ | □ |

**Scenario C4**

A 71-year-old woman presents to the emergency department 8 days after returning from visiting family in Nigeria.

She reports the relatively rapid onset of fever and abdominal pain over the prior days. She denies cough or shortness of breath. She has had no known sick contacts.

She spent the majority of her time visiting family in urban Lagos. Her only trip to the countryside was to the Lekki Conservation Center where she went birdwatching. She denies any rodent contact.

She did not attend any funerals, nor was she admitted to a hospital. She retired recently after working in the Somerville school district for many years.

Complete this scenario using**: [EVALHCID/ TRADITIONAL METHOD]**

How much time did you spend to complete this scenario?  _______ minutes

Which, HCID(s) (if any) did you evaluate the patient for?   _____________________________

In your assessment, is the patient low-risk or high-risk for the above HCID(s)?

- ​​​Low-risk (No further workup needed)
- ​​High-risk (Contact Biothreats)

|  | Strongly Disagree | Somewhat Disagree | Neutral | Somewhat Agree | Strongly Agree |
| --- | --- | --- | --- | --- | --- |
| 1. It was difficult to determine if the patient was high-risk for an HCID. | □ | □ | □ | □ | □ |
| 1. I am confident in my determination. | □ | □ | □ | □ | □ |

**Scenario D1**

A 35-year-old woman presents to the emergency department 12 days after returning from visiting family in Vietnam.

She reports diarrhea and abdominal pain that she first appreciated 3 days ago. She denies fevers or feverishness at home. She has had no known sick contacts.

She spent the majority of her time with family in rural Long An province. Her favorite aunt raises ducks for their meat and recently had to cull two sick birds.

She did not attend any funerals, nor was he admitted to a hospital. She works in Kendall Square in a biotechnology job. She does not work with any infectious agents.

Complete this scenario using: **[EVALHCID/ TRADITIONAL METHOD]**

How much time did you spend to complete this scenario?  _______ minutes

Which, HCID(s) (if any) did you evaluate the patient for?   _____________________________

In your assessment, is the patient low-risk or high-risk for the above HCID(s)?

- ​​Low-risk (No further workup needed)
- ​​High-risk (Contact Biothreats)

|  | Strongly Disagree | Somewhat Disagree | Neutral | Somewhat Agree | Strongly Agree |
| --- | --- | --- | --- | --- | --- |
| 1. It was difficult to determine if the patient was high-risk for an HCID. | □ | □ | □ | □ | □ |
| 1. I am confident in my determination. | □ | □ | □ | □ | □ |

**Scenario D2**

A 35-year-old woman presents to the emergency department 12 days after returning from visiting family in Vietnam.

She reports worsening cough and shortness of breath that she first appreciated 3 days ago. She denies fevers or feverishness at home. She has had no known sick contacts.

She spent the majority of her time with family in rural Long An province. Her favorite aunt raises ducks for their meat and recently had to cull two sick birds.

She did not attend any funerals, nor was he admitted to a hospital. She works in Kendall Square in a biotechnology job. She does not work with any infectious agents.

Complete this scenario using: : **[EVALHCID/ TRADITIONAL METHOD]**

How much time did you spend to complete this scenario?  _______ minutes

Which, HCID(s) (if any) did you evaluate the patient for?   _____________________________

In your assessment, is the patient low-risk or high-risk for the above HCID(s)?

- ​​Low-risk (No further workup needed)
- ​​High-risk (Contact Biothreats)

|  | Strongly Disagree | Somewhat Disagree | Neutral | Somewhat Agree | Strongly Agree |
| --- | --- | --- | --- | --- | --- |
| 1. It was difficult to determine if the patient was high-risk for an HCID. | □ | □ | □ | □ | □ |
| 1. I am confident in my determination. | □ | □ | □ | □ | □ |

**Scenario D3**

A 35-year-old woman presents to the emergency department 20 days after returning from visiting family in Vietnam.

She reports worsening cough and shortness of breath that she first appreciated yesterday. She denies fevers or feverishness at home. She has had no known sick contacts.

She spent the majority of her time with family in rural Long An province. Her favorite aunt raises ducks for their meat and recently had to cull two sick birds.

She did not attend any funerals, nor was he admitted to a hospital. She works in Kendall Square in a biotechnology job. She does not work with any infectious agents.

Complete this scenario using: **[EVALHCID/ TRADITIONAL METHOD]**

How much time did you spend to complete this scenario?  _______ minutes

Which, HCID(s) (if any) did you evaluate the patient for?   _____________________________

In your assessment, is the patient low-risk or high-risk for the above HCID(s)?

- ​​Low-risk (No further workup needed)
- ​​High-risk (Contact Biothreats)

|  | Strongly Disagree | Somewhat Disagree | Neutral | Somewhat Agree | Strongly Agree |
| --- | --- | --- | --- | --- | --- |
| 1. It was difficult to determine if the patient was high-risk for an HCID. | □ | □ | □ | □ | □ |
| 1. I am confident in my determination. | □ | □ | □ | □ | □ |

**Scenario D4**

A 35-year-old woman presents to the emergency department 20 days after returning from visiting family in Vietnam.

She reports worsening cough and shortness of breath that she first appreciated yesterday. She denies fevers or feverishness at home. She has had no known sick contacts.

She spent all her time in rural Ho Chi Minh City. She reports no contact with live or dead animals and mostly ate in restaurants, though she did on two occasions eat street food.

She did not attend any funerals, nor was he admitted to a hospital. She works in Kendall Square in a biotechnology job. She does not work with any infectious agents.

Complete this scenario using: **[EVALHCID/ TRADITIONAL METHOD]**

How much time did you spend to complete this scenario?  _______ minutes

Which, HCID(s) (if any) did you evaluate the patient for?   _____________________________

In your assessment, is the patient low-risk or high-risk for the above HCID(s)?

- ​​Low-risk (No further workup needed)
- ​​High-risk (Contact Biothreats)

|  | Strongly Disagree | Somewhat Disagree | Neutral | Somewhat Agree | Strongly Agree |
| --- | --- | --- | --- | --- | --- |
| 1. It was difficult to determine if the patient was high-risk for an HCID. | □ | □ | □ | □ | □ |
| 1. I am confident in my determination. | □ | □ | □ | □ | □ |
